# One Step Forward, Two Steps Back—Persistent Sex Disparities in the Cardiovascular Procedural Workforce

**DOI:** 10.1016/j.jscai.2026.105567

**Published:** 2026-08-04

**Authors:** Tamara M. Atkinson, Kimberly A. Skelding

**Affiliations:** a Department of Cardiovascular Medicine, Portland Veterans Affairs Medical Center, Portland, Oregon; b Knight Cardiovascular Institute, Oregon Health and Science University, Portland, Oregon; c Division of Cardiovascular Sciences, RWJ Barnabas Health| Jersey City Medical Center, Jersey City, New Jersey

**Keywords:** mitral TEER, sex disparities, TMVR

Elizabeth Blackwell became the first woman to matriculate into medical school in 1847, paving the way for women in medicine; however, we have been unable to maintain that momentum for women in cardiovascular invasive subspecialties. Women in medical school now comprise >50% of students, but still only 15.5% become board-certified cardiologists.^1^ It is not surprising, then, that women are even more underrepresented within cardiovascular invasive subspecialities, with 8.2% of interventional cardiologists and 10.1% of electrophysiologists being women. Women in structural heart intervention are even more underrepresented. In prior reports, transcatheter aortic valve replacement operators were noted to be 3.9% women, of which 1.5% were interventional cardiologists.^2^

In this issue of *JSCAI*, Gowda et al^3^ present important data on the sex disparities among transcatheter edge-to-edge repair (TEER)/transcatheter mitral valve replacement (TMVR) operators. With most structural heart procedures being aortic interventions, mitral valve intervention has even fewer women operators. In this report, operators who performed >10 TEER procedures and >10 TMVR procedures annually were identified using Medicare data. A total of 318 operators performed >10 TEER procedures in 2023, of which 2.2% were women. A total of 6165 TEER procedures were performed, of which 1.7% were performed by women. In 2022, a total of 10 operators performed >10 TMVR procedures, with 1 being a woman (10%). Most states within the United States (88%) did not have women TEER operators.

Women operators in interventional cardiology and structural intervention are lagging behind the surge in procedural volume. While transcatheter aortic and mitral interventional volume continues to rise, this study, as well as previous publications,^2^ shows that the number of women operators in structural heart disease is stagnant. How can we do better to sustain our current operators as well as support and encourage future women operators?

First, we must understand the barriers to recruiting and retaining women in the field of interventional and structural cardiology. The American College of Cardiology (ACC) conducted a professional life survey in 1996 that highlighted radiation exposure, family responsibilities, unequal compensation, and lack of career advancement opportunities as some sex-specific concerns.^4^ Twenty years later, women continue to face similar challenges, which were evident in the recent WomEn in interventional Cardiology: A qualitative REsearch (WECARE) study. The WECARE study evaluated the career experiences and challenges faced by practicing women interventional cardiologists.^5^ Similar themes emerged, which included barriers to career entry and promotion, family responsibilities, gender bias, variable mentorship, and radiation issues. On a positive note, this study highlighted the career satisfaction and commitment to interventional cardiology as well as the pivotal role that mentorship from both men and women had in their career path.

We have built programs within our professional societies to support women, such as ACC Women in Cardiology, Society for Cardiovascular Angiography & Interventions (SCAI) Women in Innovations (WIN), and European Association of Percutaneous Cardiovascular Interventions women’s committee, but we have more to do on this front. First, expanded mentorship and sponsorship of women into cardiovascular fellowships will promote more women into invasive subspecialties. It is encouraging that there has been an increase in women in interventional cardiology fellowships, with 20% of fellows being women^5^; however, we know that women interventionalists are leaving the field more quickly than their male colleagues, with an annual probability for leaving of 21.1% for women and 14.9% for men.^6^

Systemic changes within institutions and professional societies are still needed to improve the recruitment and retention of women in cardiovascular invasive subspecialties. Potential solutions have been proposed in these prior reports, which are summarized below:^1^^,^^3^^,^^5^

1. Mentorship and sponsorship: across professional societies and within our institutions and communities, exposure to and visibility of women operators are important factors for how medical students choose an interventional career. Male mentors can positively engage female students in career choices and are vital to making a change in medical specialty numbers.
2. Radiation protection: while radiation issues are improving with new radiation protection devices, they are still not universally available. As a profession, we need to mandate that newer cardiac catheterization lab builds include radiation protection devices.
3. Work-life balance: employers need to empower and support employees to have flexible work hours or part-time schedules. Both women and men have different needs throughout their careers. Survey data have shown that women bear more of the burden for childcare and parental care for aging parents, which should not stall their career trajectory.
4. Standardize parental leave: this benefit should be hardwired into employment contracts rather than a negotiable timeframe.
5. Leadership opportunities: support and encourage the advancement of appropriate women candidates into leadership roles. Support women in national meeting presentations, live case operator roles, national committees, and board positions.
6. Advancement metrics: provide training for women leaders and their leadership teams to combat implicit bias when measuring skillsets across the sexes. There should be no double standards. Value needs to be placed on leadership skills that diverse team members, including all underrepresented minorities, bring to the table rather than measuring a one-size-fits-all leadership style. The diversity these leaders bring to the field of cardiology should be celebrated and not criticized. An accepting culture and equity in professional practice are frequently cited as vitally important to women seeking leadership, advanced fellowships, and procedural positions.^7^ The environment needs to support this and have a zero-tolerance policy for discriminatory practices.
7. Training programs: consider fast-track fellowships for interventional and structural cardiology, which will increase numbers across the board. SCAI should consider a WIN structural fellowship similar to the SCAI-WIN CHIP fellowship to provide an environment of success.^3^

In the words of “Rosie the Riveter,” who empowered the workforce of the 1940s, “We can do it!” The continued change that needs to occur will require both men and women to support, encourage, and remove barriers from bias that occur for our women colleagues. Changes are needed on an educational, institutional, and community-wide basis to mentor future women in invasive cardiovascular specialties.

## CRediT authorship contribution statement

**Tamara M. Atkinson:** Writing – original draft, Writing – review & editing. **Kimberly A. Skelding:** Writing – review & editing.

## Declaration of competing interest

Kimberly Skelding is involved in research and proctoring at Medtronic. Tamara Atkinson reported no financial interests.
